# Very Low Efficiency of Direct Reprogramming of Astrocytes Into Neurons in the Brains of Young and Aged Mice After Cerebral Ischemia

**DOI:** 10.3389/fnagi.2019.00334

**Published:** 2019-12-03

**Authors:** Andrei Gresita, Daniela Glavan, Ion Udristoiu, Bogdan Catalin, Dirk M. Hermann, Aurel Popa-Wagner

**Affiliations:** ^1^Menzies Health Institute of Queensland, Griffith University, Gold Coast Campus, Gold Coast, QLD, Australia; ^2^Chair of Vascular Neurology, Dementia and Ageing Research, Department of Neurology, University of Duisburg-Essen, University Hospital Essen, Essen, Germany; ^3^Department of Psychiatry, University of Medicine and Pharmacy Craiova, Craiova, Romania; ^4^Center of Clinical and Experimental Medicine, University of Medicine and Pharmacy, Craiova, Romania

**Keywords:** aging, cerebral ischemia, therapy, glial scar, genetic conversion

## Abstract

After cerebral ischemia, the ratio between astroglial cells and neurons in the neurovascular unit is disrupted in the perilesional area. We hypothesized that restoring the balance within the neurovascular unit may lead to an improved neurorestoration after focal ischemia. Recently, an innovative technology has been invented to efficiently convert proliferating astroglial cells into neurons in the injured young brain. However, the conversion efficacy of this technology has not been explored in the post-stroke brains of the aged rodents. To this end, we used a retroviral delivery system encoding the transcription factor *Ngn2* alone or in combination with the antiapoptotic factor *Bcl-2* to target proliferating astrocytes in the neocortex of young and aged mice after cerebral ischemia. Successful direct *in vivo* reprogramming of reactive glia into neuroblasts and mature neurons was assessed by cellular phenotyping. We found that the conversion efficacy of proliferating astrocytes into neurons after cerebral ischemia in young and aged mice is disappointingly low, most likely because the therapeutic vectors carrying the conversion gene are engulfed by phagocytes shortly after intracortical administration. We conclude that other viral vectors and combinations of transcription factors should be employed to improve the efficacy of glia-to-neuron conversion after stroke in young and aged rodents.

## Introduction

Ischemic stroke is an acute disease that often results in severe long-term consequences such as physical disability, depression, cognitive decline or even dementia.

Brain infarction has a strong age dependency both in men and women. Statistically, the prevalence of stroke in the patients over 75 years is 50% but decreases to 30% in the elderly over 85 years (Roger et al., 2012). In addition, infarct development is age-dependent, suggesting that old age may predict outcome in acute stroke (Ay et al., 2005).

The majority of the drugs tested showed safety and therapeutic efficacy in young animal models. However, most of the drugs tested in young animals failed to show efficacy when tested in clinical trials. One major reason for this translational failure may be the use of young animals instead of aged subjects in stroke research (Popa-Wagner et al., 2014; Hermann et al., 2019). Luckily, in the last several years there has been an increased awareness of this shortcoming paralleled by an increased number of studies done in aged rodents as recommended by the Stroke Progress Review Group (Fisher et al., 2009).

Direct astroglial reprogramming to neurons using viral vectors carrying transcription factors such as Sox2 (Niu et al., 2013; Heinrich et al., 2014), Ngn2 (Grande et al., 2013; Heinrich et al., 2014; Gascón et al., 2016), or Ascl1 (Torper et al., 2015; Rivetti di Val Cervo et al., 2017) provides a completely new strategy for neurorestoration after stroke. During the acute phase of stroke, neurons are lost in the infarcted area and to some extent in the perilesional area while astroglia cells proliferate and migrate into the lesioned area and disrupt the neurovascular cell balance. This is more evident in the aged brains which mount a fulminant astroglial reaction and an inflammatory response to cerebral ischemia in the lesioned area (Popa-Wagner et al., 2007b). The astroglial proliferative response to the acute ischemic event leads to the early build-up of a growth-inhibitory fibrotic scar composed mainly of activated astrocytes surrounded by a large number of activated and phagocytosing microglia cells that may last up to 3 months (Sofroniew and Vinters, 2010; Robel et al., 2011; Ge et al., 2012; Tsai et al., 2012; Burda and Sofroniew, 2014).

Therefore, the advantage of converting reactive glial cells into neurons in the perilesional area is 2-fold: (i) prevent the build-up of a growth-inhibitory glial scar; and (ii) restore the cellularity balance within the neurovascular unit in the perilesional area by reducing the number of activated astrocytes by conversion into neuronal cells.

However, given the poor translatability of drug efficacy tested in young subjects in the older subjects, it is of interest to test the efficacy of this new approach in the post-stroke aged rodent brain. To this end, we used a retroviral delivery system encoding the transcription factor *Ngn2* alone or in combination with the anti-apoptotic factor *Bcl-2*, to target proliferating astrocytes in the neocortex of young and aged mice after cerebral ischemia.

## Materials and Methods

### Animals and Experimental Design

For our study, we used 29 mice divided as follows: 16 young (3 months) C5BL6 mice and 13 old (18 months) transgenic TYFE mice, all kept under strict laboratory conditions. TgN(Thy1.2-EYFP) are transgenic animals obtained after cloning the open reading frame of HcRed1 into the XhoI site of the Thy1.2-minigene. The end result is transgenic mice expressing yellow fluorescent protein (EYFP) in neurons. The animals were originally described by Prof. Frank Kirchhoff (Hirrlinger et al., 2005; Winter et al., 2007). However, the animals used in this study were bred, selected by genotyping and provided by Dr. BC, University of Medicine and Pharmacy Craiova.

All experiments were performed in accordance with ARRIVE Guidelines for the Care and Use of Laboratory Animals, and animal procedures were approved (#111-18-10-2018) by the University of Medicine and Pharmacy Craiova.

### Surgery

Mice were anesthetized with 100 mg/kg ketamine and 10 mg/kg xylazine (ip) and the head placed in a stereotaxic device. After craniotomy, the MCA was exposed and occluded using a fine soldering iron directly through the dura. Saline was applied to the area throughout the procedure to prevent heat injury to the brain surface. The muscle and soft tissue were replaced and the skin was closed using 5–0 nylon suture. Buprenorphine was given sc twice, every 6 h after surgery at a dose of 0.3 mg/kg for peri-operative pain relief. Moisted food was provided for the first 3 days post-surgery.

### Treatment

The young C57BL6 mice were divided into the following groups: (1) the Ngn2 treatment groups (*N* = 4 mice/group) received 1 μl of RV-Ngn2-RFP, given, under stereotaxic guidance, intracortically at three different locations in the area proximal to the occluded artery immediately after stroke. In preliminary experiments, we tested whether administration of a mix consisting of 0.5 μl RV-Ngn2-RFP and 0.5 μl RV-Bcl2-GFP would increase the rate of conversion into neuronal cells. The results did not show any improvement and this treatment was discontinued for the young mice. The aged transgenic TYFE mice (*N* = 5 mice/group) received 1 μl of a mix consisting of 0.5 μl RV-Ngn2-RFP and 0.5 μl RV-Bcl2-GFP or vehicle (*N* = 3), immediately after stroke. In order to increase the number of conversions to NeuN-positive neurons, 200 μl of a-Tocopherol (1 mg/ml) diluted in corn oil, was administered through oral gavage at day 2 after stroke (Gascón et al., 2016). The viral constructs were a gift from Dr. Magdalena Goetz, Helmholtz Center, Munich, Germany.

**Figure 1:**
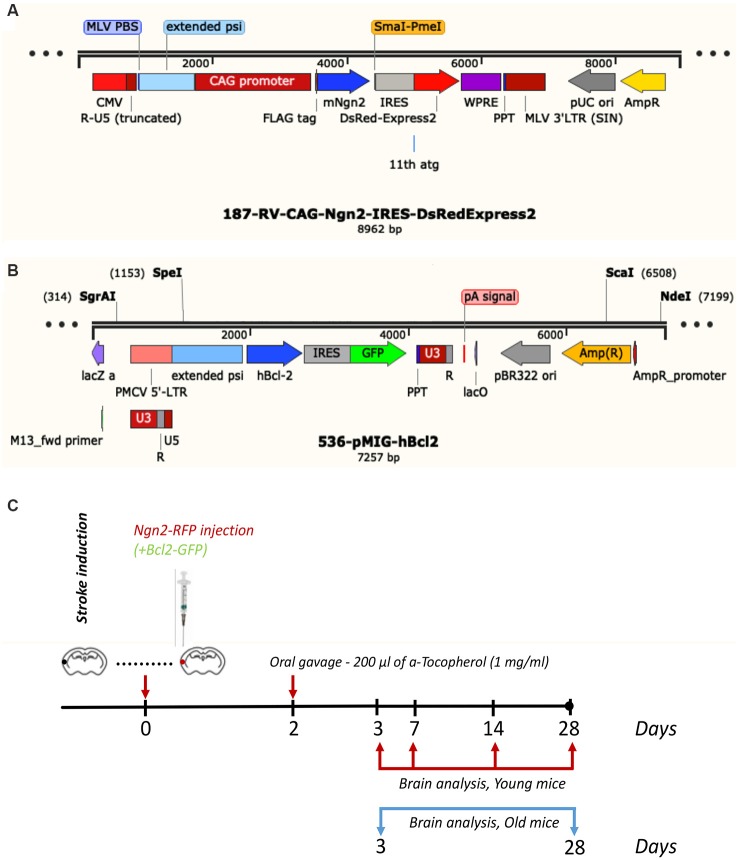
Viral constructs **(A,B)** and experimental design **(C)**.

Subsequent to survival times of 3-, 7-, 14- and 28 days, the young mice were anesthetized with a mix of ketamine and xylazine and perfused with phosphate buffer saline (PBS) followed by 4% freshly depolymerized paraformaldehyde in 50 mM PBS. The old transgenic mice were sacrificed at 3- and 28 days post-stroke. The experimental design and the viral constructs are shown in Figure 1. The brain was removed, post-fixed in 4% paraformaldehyde for 24 h, cryoprotected first in 15% glycerol/50 mM PBS, flash-frozen in isopentane, and stored at −70°C until cryostat sectioning.

### Cellular Phenotyping by Immunohistochemistry

Cryostat, 25 μm free-floating sections were fixed in 4% paraformaldehyde/50 mM for 15 min and then washed extensively with PBS. For phenotyping, the tissue was incubated with: (i) macrophages marker, rabbit anti-ED1 (1:1,000, Abcam, UK); or (ii) the neuronal nuclei marker, rabbit anti-NeuN (1:1,000, Millipore, Germany); or (iii) the astrocytic marker, rabbit anti-GFAP (1:1,000, Abcam, UK) at 4°C overnight. The next day, sections were rinsed with PBS and incubated with Alexa Fluor^®^ 488 goat anti-rabbit IgG or Alexa Fluor^®^ 647 goat anti-rabbit IgG for the aged mice brains. After final rinsing, sections were brought to Superfrost Plus slides and mounted using PVA/DABCO-containing medium.

### Cell Quantitation

A quantitative estimate of the number of ED1-, GFAP-, and NeuN-immunopositive cells was obtained by counting the cells in 25-mm-thick sections in area units measuring 250 × 250 μm, using a “random-systematic” protocol (random start point for a systematic series of every 10th section through the infarcted volume) as previously described (Popa-Wagner et al., 2007a). The area occupied by cells of interest was 30% of the total stained area. The relative mean number of immunopositive cells was then calculated per group, time point, and age by multiplying the number of cells per section × 3.3 (the counting boxes that were quantitated covered one-third of the lesioned area of each section) times the section interval of 10. Finally, the percentage of double-labeled cells was calculated by dividing the number of double-labeled cells by the total number of cells.

### Statistical Analysis

Statistical analysis was performed using GraphPad Prism software (Version 7.0). Statistical significance was determined by one-way ANOVA with Tukey’s *post hoc* test for multiple group comparisons. All results are given as the means ± SD. The results were considered statistically significant at a *p*-value of < 0.05.

## Results

**Figure 2:**
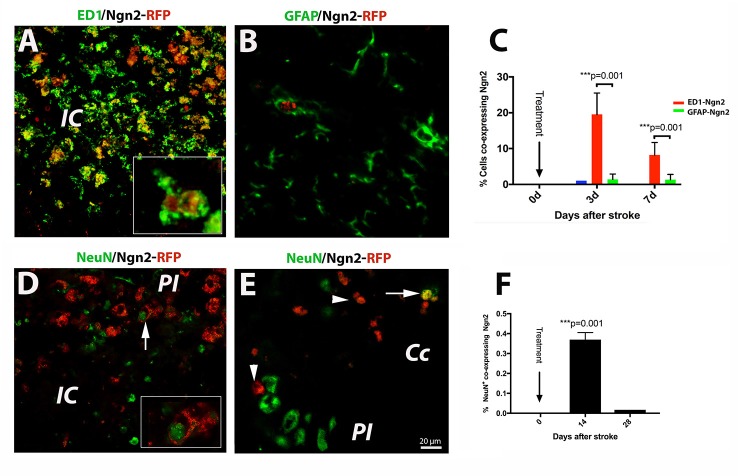
(*Upper panel*) *In situ* reprogramming of proliferating ED1- and GFAP-positive cells by retroviral expression of Ngn2-RFP in the brains of young mice. By immunofluorescence, the large majority (19.4%, **B**) of the Ngn2-RFP+ cells were macrophages cells at day 3 after stroke (**A**; and inset). Thereafter, by day 7 post-stroke, the number of cells co-expressing ED1 and Ngn2-RFP+ dropped to 8% **(C)**. Only a very small fraction of cells (less than 1%) co-expressed the astrocytic marker GFAP and Ngn2-RFP in the peri-infarcted area **(B)**. (*Lower panel*) Phenotyping and quantification of transduced cells in young mice. The conversion efficiency into neurons in the infarcted area of young mice was very low, about 0.35% (**D** and inset; **F**). In corpus callosum, we found, on rare occasions, double-labeled cells which were, based on morphology and localization in the corpus callosum, most likely, of hematogenous origin (**E**, arrow). Scale bar, 20 μm. *N* = 4 mice/group. ***P < 0.001, by one-way ANOVA with Tukey’s *post hoc* test for multiple group comparisons.

Of the previously tested transcription factors used to convert astrocyte into neurons, we chose Neurog2, as it converts astrocytes into glutamatergic neurons after stab injury *in vivo* (Gascón et al., 2016). Previous reports indicated that following a stab injury to the brain, most of the infected cells the retrovirus carrying the Neurog-2-IRES-RFP sequence were GFAP+ astrocytes. To our surprise, however, following brain injury due to focal ischemia, the large majority (19.4%, Figure 2A) of the RFP+ cells were macrophages at day 3 after stroke (Figure 2A; and inset). Thereafter, by day 7 post-stroke the number of cells co-expressing ED1 and Ngn2-RFP+ dropped to 8% (Figure 2C). Only a very small fraction of cells (1%) co-expressed the astrocytic marker GFAP and Ngn2-RFP in the peri-infarcted area (Figures 2B,C).

Recently, it was reported that about 75% of retrovirus-infected cells had turned into NeuN+ neurons at day 10 after stab injury to the brain (Gascón et al., 2016). In our experiments using the mouse model of focal ischemia which causes a much more severe injury, the conversion efficiency in the infarcted area was much lower, about 0.35% (Figure 2D and inset, Figure 2F). In corpus callosum, we found, on rare occasions, double labeled cells, which were based on nuclear morphology and localization, most likely of blood-born cells (Figure 2E, arrow).

**Figure 3:**
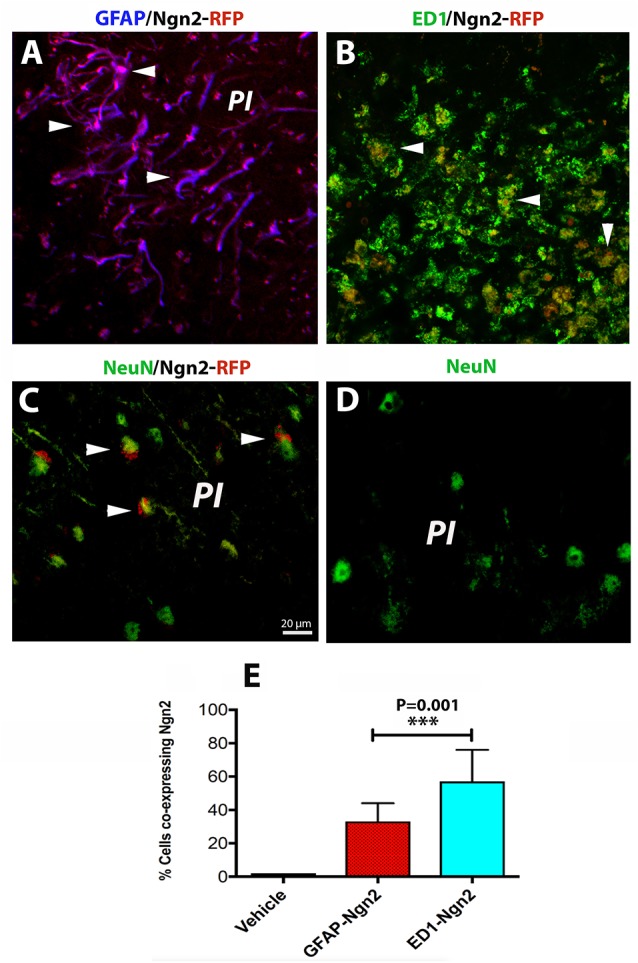
In the aged, post-stroke brains of the transgenic mice, astroglia was highly proliferative **(A)**. In the aged brains at day 3 after stroke (*N* = 5 mice), the fraction of infected astrocytes in the peri-infarcted with the retrovirus carrying the Ngn2-RFP sequence was high (57%) in the aged brain **(E)**. At the same time point, the number of ED1-RFP co-labeled cells was 33% (**B**, arrow heads; **E**). At day 28, in the lesioned brains of aged post-stroke transgenic mice displaying GFP-neurons, we found indeed a large number of co-labeled Ngn2-RFP/NeuN-GFP cells. However, a closer examination of their nuclei revealed that Ngn2-RFP expression was confined to the NeuN-GFP-labeled neuronal nuclei (**C**, arrowheads). Animals treated with the vehicle (*N* = 3) showed NeuN-GFP neuronal nuclei only **(D)**. Scale bar, 20 μm. ***P < 0.001, by one-way ANOVA with Tukey’s *post hoc* test for multiple group comparisons.

The risk of having a stroke event doubles each decade after the age of 55 (Kelly-Hayes, 2010). Therefore, there is of great interest to develop neurorestorative therapies of stroke which occurs mostly in elderly people. As expected, astroglia was highly proliferative after cerebral ischemia (Figure 3A) and induced a highly inflammatory environment, especially in the brains of the aged rats (Sandu et al., 2016). On day 3 after stroke, the fraction of infected astrocytes with the retrovirus carrying the Ngn2-RFP sequence in the aged brain was high (57%, on average, Figures 3A,E) in the peri-infarcted area as compared to young animals. At the same time point, the number of infected macrophages with RV-Ngn2-RFP was lower (33%) as compared to the young, non-transgenic mice (Figures 3B,E). At day 28, in the lesioned brains of the aged post-stroke transgenic mice displaying GFP-neurons, we found occasionally clusters of co-labeled Ngn2-RFP/NeuN-GFP cells. However, after a careful examination of their nuclei, we concluded that Ngn2-RFP expression was adjacent to the NeuN-GFP-labeled neuronal nuclei suggesting that the retrovirus has eventually infected also injured but still viable aged neurons but the Ngn2-RFP sequence was not fully expressed (Figure 3C, arrowheads). Vehicle-treated animals showed the NeuN-GFP neuronal nuclei only (Figure 3D).

## Discussion

Treatments for acute stroke patients are still very limited to invasive treatment methods such as intra-arterial thrombectomy and artery recanalization using thrombolysis which can be used within the first hours after the stroke event. However, these procedures are not useful for tissue recovery during the rehabilitation phase.

The development of a growth-inhibitory astrocytic scar after cerebral ischemia may impede long-term regenerative events in the injured brain. Recently, new approaches have been developed to transform the inhibitory scar tissue into a more permissive environment allowing neuronal regeneration and axonal growth by direct conversion of astrocytes into neurons. In this work, we used a retroviral delivery system encoding the transcription factor *Ngn2* alone or in combination with the anti-apoptotic factor *Bcl-2* to target proliferating astrocytes in the neocortex of young and aged mice after cerebral ischemia. We found that the conversion efficacy of proliferating astrocytes into neurons after cerebral ischemia in young and aged mice is disappointingly low, most likely because the therapeutic vectors carrying the conversion gene are engulfed by phagocytizing cells shortly after the intracortical administration.

Therefore, the first step to improve this technology would be to increase the efficiency of viral conversion of proliferating astroglia into neurons and thus restore, at least in part, the cell balance in the injured area. However, the efficiency of neuronal reprogramming is severely limited by cell death due to apoptosis during transcriptional conversion which peaks at the time of fate conversion. For this reason, a second retroviral vector encoding the anti-apoptotic factor, Bcl2 has been used (Gascón et al., 2016).

A third limiting factor of the genetic conversion turned out to be the fulminant inflammatory response mounted by microglia cells in response to cerebral ischemia, especially in the aged brains (Sandu et al., 2016). Subtoxic insults such as inflammation may cause the injured but still viable neurons to express, the “eat-me” signal phosphatidylserine (PS) on their surface. When migrating microglia detect the “eat-me” signals complete or partial engulfment of these stressed neurons follows. This process has been coined primary phagocytosis or “phagoptosis” (Fuhrmann et al., 2010; Neher et al., 2011; Brown and Neher, 2014). At the same time, however, the phagocytes will engulf the therapeutic vector, too. It is, therefore, a challenge to develop stroke therapies that limit the capability of phagocytes to engulf altogether stressed but still viable neurons and the therapeutic vectors in the injured area after cerebral ischemia.

In the clinic, post-stroke functional recovery in patients is efficiently stimulated using physiotherapy, especially in the young subjects. In animal models, the mechanism behind functional recovery after stroke has been associated with recruitment of neural circuits in the perilesional area and unmasking of existing, but latent circuits in the contralateral hemisphere (Dijkhuizen et al., 2001). In humans, the post-stroke deficit may be compensated by regression of perilesional inhibition and from remote intracortical disinhibition (Alia et al., 2017). However, dead tissue in the infarct core and to some extent in the perilesional area, cannot be replaced by physiotherapy alone. Therefore, replacement of lost tissue has been attempted by numerous approaches including, fetal tissue transplantation, stem cell transplantation including iPS or, more recently by 3D printing strategies (for a review, see Wechsler et al., 2018). A quite different approach is to convert genetically the excessive number of astroglia cells in the perilesional area into neuronal tissue using viral vectors (Guo et al., 2014). This technology, however, has to be optimized by addressing key translational aspects including safety and efficiency.

## Conclusion

Post-stroke brain repair by genetic conversion *in situ* reprogramming of reactive astroglia into functional neurons in the perilesional area is a completely new approach of tissue replacement. However, the conversion efficacy of proliferating astrocytes into neurons after cerebral ischemia in young and aged mice is disappointingly low and could be due to: (i) cell death at the time of fate conversion; and (ii) fulminant inflammatory response to cerebral ischemia resulting in phagocytosis of the therapeutic vector shortly after intracortical administration.

## Data Availability Statement

The datasets generated for this study are available on request to the corresponding author.

## Ethics Statement

The animal study was reviewed and approved by University of Medicine and Pharmacy, Craiova, Romania.

## Author Contributions

AP-W and DH: conceptualization, formal analysis, supervision and funding acquisition. AG, DG, and IU: methodology. AP-W, DG, and IU: writing—original draft. AP-W: project administration. AG: surgery. AG, DG, and BC: immunohistostaining and figures drawing.

## Conflict of Interest

The authors declare that the research was conducted in the absence of any commercial or financial relationships that could be construed as a potential conflict of interest.
